# Type-specific prevalence of human papillomavirus in women screened for cervical cancer in Labrador, Canada

**DOI:** 10.3402/ijch.v72i0.19743

**Published:** 2013-02-21

**Authors:** Alberto Severini, Ying Jiang, Paul Brassard, Howard Morrison, Alain A. Demers, Elizabeth Oguntuase, Muna Al-Rushdi, Felicia Preston, Samuel Ratnam, Yang Mao

**Affiliations:** 1 National Microbiology Laboratory, Public Health Agency of Canada, Winnipeg, MB, Canada; 2 Department of Medical Microbiology and Infectious Diseases, University of Manitoba, Winnipeg, MB, Canada; 3 Centre for Chronic Disease Prevention and Control, Public Health Agency of Canada, Ottawa, ON, Canada; 4 Division of Clinical Epidemiology, McGill University Health Center, Montreal, QC, Canada; 5 Centre for Communicable Diseases and Infection Control, Public Health Agency of Canada, Ottawa, ON, Canada; 6 Department of Community Health Sciences, University of Manitoba, MB Canada; 7 Labrador-Grenfell Health, Happy Valley-Goose Bay, NL, Canada; 8 Faculty of Medicine, Memorial University, St. John's, NL, Canada

**Keywords:** human papillomavirus, cervical cancer, aboriginal populations, prevalence

## Abstract

**Background:**

A higher incidence of cervical cancer and human papillomavirus (HPV) infection has been reported in northern Canada and in First Nation, Métis and Inuit women, with some evidence to suggest that the HPV type distribution in these populations may be different from the rest of Canada.

**Objective:**

The objective of this study was to measure the HPV type prevalence in Labrador women to determine if significant differences in HPV types could reduce the effectiveness of HPV vaccination.

**Design:**

The prevalence of HPV types was determined in 1,370 women presenting for routine pap screening in Labrador between February and November 2010. Cervical cytology and HPV genotyping were performed on the same liquid-based cytology specimens.

**Results:**

The overall prevalence of HPV was 21.4%; cytological abnormalities were found in 8.8% of the participants. HPV 16 and 18 were the most common high-risk HPV types. These two types were found in 52.4% of high-grade lesions. The prevalence in HPV infections was comparable across the Labrador regions.

**Conclusions:**

The present results support the potential effectiveness of the HPV immunization program in Labrador.

Every year in Canada, approximately 1,300 women are diagnosed with cervical cancer and 450 die from it (1). Aboriginal women living in Canada (Inuit, Métis and First Nations) have been reported to suffer a higher burden of cervical cancer (2–5). Not surprisingly, higher prevalence of HPV infection has also been reported in these women (6–8), with one study suggesting that the distribution of HPV types in Inuit populations may differ compared to the rest of Canada (6).

Over 50 different human papillomavirus (HPV) types infect the anogenital tract, some of which are referred to as high-risk (HR) types for cancer (9). These HR-HPV types are a necessary cause for developing cervical cancer and are involved in a sizable proportion of other anogenital and oropharyngeal malignancies. In particular, HPV types 16 and 18 have been found in over 70% of cervical cancers worldwide (10,11), 90% of HPV-positive anal cancers and 10–60% of oropharyngeal malignancies (12). These findings have been confirmed in many studies and summarized in recent meta-analyses (11,13).

Labrador is a vast region of 294,330 km^2^ inhabited by approximately 26,000 people with a predominantly Aboriginal population. The Aboriginal population living in Labrador is composed of Métis, Inuit and First Nations. We were interested in determining the distribution of HPV types in women living in Labrador and carried out a surveillance study using a convenience sample of women attending routine cervical screening.

## Methods

Women in Labrador who presented for routine Papanicolaou (Pap) test screening in 17 Labrador communities between February 2010 and November 2010 were introduced to the study. Liquid-based cytology (LBC, SurePath^®^, BD Diagnostics) samples were collected as per standard practice and shipped to the Newfoundland Public Health Laboratory in St John's. Specimens were aliquoted for HPV genotyping and shipped to the National Microbiology Laboratory (NML) in Winnipeg, Manitoba. Cytology was carried out according to provincial guidelines and practices at the Eastern Health Cytology Laboratory in St John's. Cytology and HPV testing were carried out independently in a blinded fashion.

Cytology results were reported according to the Bethesda system (14) (normal (including benign), atypical squamous cells of undetermined significance (ASCUS), low-grade squamous intraepithelial lesion (LSIL), atypical squamous cells in which high-grade lesion could not be excluded (ASC-H), atypical glandular cells (AGC) and high-grade squamous intraepithelial lesion (HSIL)). The cytological outcomes ASC-H, AGC and HSIL were combined in the category “high-grade lesions” and the cytological outcomes ASCUS and LSIL were combined in the category “low-grade lesions”. HPV types were grouped into species, according to the most recent classification (15).

HPV typing was performed at the NML using an “in-house” Luminex-based method that detects 45 HPV types (16). These include 23 of the 25 types defined as carcinogenic by the International Agency for Research on Cancer (IARC Group 1: types 16, 18, 31, 33, 35, 39, 45, 51, 52, 56, 58, 59), and probably or possibly carcinogenic (IARC Group 2: types 26, 53, 66, 67, 68, 70, 73, 82, 30, 69, 85) (9), as well as 22 low-risk (LR) or unknown risk types, including HPV 6, 11, 13, 32, 40, 42, 43, 44, 54, 61, 62, 71, 72, 74, 81, 83, 84, 86, 87, 89, 90, and 91. HPV genotyping was carried out as previously described (17,18).

Cytology and HPV typing results were linked to demography data using a study identification number. For women who had more than one Pap test over the study period, results from the first specimen were taken. Many of the 17 communities in this study have a small population and therefore, in order to build up sufficient numbers for statistical analyses and to avoid identification of specific communities, the participants were grouped in three geographical regions, namely the North coast (Nain, Hopedale, Makkovik, Postville, Rigolet and Natuashish), the South coast (Cartwright, Black Tickle, Charlottetown, St. Lewis, Port Hope Simpson, Mary's Harbour, L'Anse au Loup and Forteau) and the Central region (Happy Valley-Goose Bay, Sheshatshiu and North West River). According to the 2006 Canadian census, approximately 40% of the population in the Central region self-identified as Aboriginal, while at least 87% self-identified as Aboriginal in the North coast and South coast. Univariate comparisons were made using χ^2^. Prevalence rates are presented in percentages. Regions were compared using logistic regression and the p-values reported are the type 3 analysis of effect. Analyses were performed using SAS^®^.

Ethics approval was obtained from the Public Health Agency of Canada ethics boards. Labrador-Grenfell Health facilitated the project and community stakeholders reviewed the manuscript before being submitted for publication.

## Results

Of the 1,370 women that were enrolled in the study, 293 (21.4%) were infected with at least one HPV type and 218 (15.9%) tested positive for at least one HR-HPV type. The infected women had a total of 398 HPV infections; 77 of HPV-positive women had multiple infections. The age of the participants ranged from 13 to 86 years (mean: 36.9; median: 35). Overall, 90.4% of participants had a normal cytology, 3.5% had an ASCUS, 3.7% a LSIL, 1.5% a high-grade lesion (AGC, ASC-H, HSIL) and 0.8% had an unsatisfactory Pap test.

At least one HR-HPV type in the IARC Group 1 (carcinogenic) was detected in 12.5% of participants; 4.6% were positive for at least one IARC Group 2 type (probably or possibly carcinogenic); and 5.6% tested positive for multiple HPV infections (Table I). Study participants from the three geographical regions had comparable risk of having a HPV infection with any HPV types or with a HR-HPV type (Table II). The probability of being diagnosed with a cervical abnormality was comparable in the three geographical areas.

**Table I:** Cytological outcomes and HPV types by age and Labrador region

		Cytological outcomes						HPV infections																		
				
		Normal		Abnormal		High-grade^a^		Any		Multiple infections		Group 1^b^		Multiple Group 1		Group 2^c^		Multiple Group 2		HPV 16/18		HPV 31		HPV 16		Total
														
Geography	Age	n	%	n	%	n	%	n	%	n	%	n	%	n	%	n	%	3	2.0	n	%	N	Col%	N	Col%	N
Central region	<25	111	75.0	36	24.3	3	2.0	70	47.3	30	20.3	49	33.1	15	10.1	20	13.5	1	1.2	21	14.2	3	2.0	12	8.1	148
	25–29	75	89.3	9	10.7	1	1.2	18	21.4	5	6.0	8	9.5	0	0.0	5	6.0	0	0.0	3	3.6	0	0.0	2	2.4	84
	30–39	172	93.0	12	6.5	3	1.6	36	19.5	7	3.8	14	7.6	2	1.1	11	5.9	0	0.0	7	3.8	1	0.5	5	2.7	185
	≥40	285	95.3	14	4.7	5	1.7	34	11.4	2	0.7	18	6.0	0	0.0	3	1.0	4	0.6	6	2.0	3	1.0	5	1.7	299
	All	643	89.8	71	9.9	12	1.7	158	22.1	44	6.1	89	12.4	17	2.4	39	5.4	1	0.7	37	5.2	7	1.0	24	3.4	716
North coast	<25	120	81.1	26	17.6	1	0.7	62	41.9	20	13.5	37	25.0	7	4.7	11	7.4	0	0.0	16	10.8	2	1.4	12	8.1	148
	25–29	66	89.2	7	9.5	1	1.4	22	29.7	3	4.1	12	16.2	0	0.0	6	8.1	0	0.0	6	8.1	0	0.0	6	8.1	74
	30–39	76	96.2	1	1.3	1	1.3	9	11.4	3	3.8	4	5.1	1	1.3	2	2.5	1	0.8	1	1.3	0	0.0	0	0.0	79
	≥40	118	95.9	5	4.1	1	0.8	11	8.9	3	2.4	7	5.7	1	0.8	3	2.4	2	0.5	4	3.3	0	0.0	2	1.6	123
	All	380	89.6	39	9.2	4	0.9	104	24.5	29	6.8	60	14.2	9	2.1	22	5.2	0	0.0	27	6.4	2	0.5	20	4.7	424
South coast	<25	30	85.7	5	14.3	2	5.7	15	42.9	4	11.4	10	28.6	2	5.7	2	5.7	0	0.0	6	17.1	1	2.9	5	14.3	35
	25–29	9	100.0	0	0.0	0	0.0	2	22.2	0	0.0	1	11.1	0	0.0	0	0.0	0	0.0	1	11.1	0	0.0	0	0.0	9
	30–39	48	92.3	4	7.7	2	3.8	10	19.2	0	0.0	9	17.3	0	0.0	0	0.0	0	0.0	2	3.8	1	1.9	1	1.9	52
	≥40	129	96.3	1	0.7	1	0.7	4	3.0	0	0.0	2	1.5	0	0.0	0	0.0	0	0.0	0	0.0	0	0.0	0	0.0	134
	All	216	93.9	10	4.3	5	2.2	31	13.5	4	1.7	22	9.6	2	0.9	2	0.9	4	1.2	9	3.9	2	0.9	6	2.6	230
Labrador	<25	261	78.9	67	20.2	6	1.8	147	44.4	54	16.3	96	29.0	24	7.3	33	10.0	1	0.6	43	13.0	6	1.8	29	8.8	331
	25–29	150	89.8	16	9.6	2	1.2	42	25.1	8	4.8	21	12.6	0	0.0	11	6.6	0	0.0	10	6.0	0	0.0	8	4.8	167
	30–39	296	93.7	17	5.4	6	1.9	55	17.4	10	3.2	27	8.5	3	0.9	13	4.1	1	0.2	10	3.2	2	0.6	6	1.9	316
	≥40	532	95.7	20	3.6	7	1.3	49	8.8	5	0.9	27	4.9	1	0.2	6	1.1	6	0.4	10	1.8	3	0.5	7	1.3	556
	All	1,239	90.4	120	8.8	21	1.5	293	21.4	77	5.6	171	12.5	28	2.0	63	4.6	3	2.0	73	5.3	11	0.8	50	3.6	1,370

a Includes atypical squamous cells, unable to exclude high-grade intraepithelial lesion (ASC-H), atypical glandular cells (AGC) and high-grade intraepithelial lesion (HSIL).

b IARC Group 1: HPV 16, 18, 31, 33, 35, 39, 45, 51, 52, 56, 58, 59.

c IARC Group 2: HPV 26, 53, 66, 67, 68, 70, 73, 82, 30, 69, 85.

**Table II:** Risk of cervical abnormality, any type of HPV infection and infection with HR-HPV by region (models are adjusted for age)

	Any cervical abnormality		Any HPV infection		High-risk HPV infection	
			
Geography	OR	95% CI	OR	95% CI	OR	95% CI
Central region	1.00	–	1.00	–	1.00	–
North coast	0.69	0.45–1.05	0.85	0.63–1.15	0.86	0.60–1.25
South coast	0.51	0.26–1.03	0.69	0.44–1.07	0.96	0.57–1.60

Infections with species A9, which includes types closely related to HPV 16, were the most frequent (32.2% of infections), including HPV 16 (12.3%), HPV 33 (4.5%), HPV 58 (3.5%) and HPV 67 (3.8%) (Table III). Species A7, which includes types closely related to HPV 18, was the second most common species (18.8% of infections), of which HPV 18 (6.3%) was the most prevalent type. HPV types belonging to species A9 and A7 account for almost all cases of cervical cancer (15).

**Table III:** Distribution of HPV species and types in Labrador (Canada)

Species	HPV type	n	%	Species	HPV type	n	%
A1	32	5	1.3	A8	40	4	1.0
	42	20	5.0		43	1	0.3
	Total	25	6.3		91	0	0.0
A3	61	1	0.3		Total	5	1.3
	62	18	4.5	A9	16	49	12.3
	72	9	2.3		31	11	2.8
	81	15	3.8		33	18	4.5
	83	6	1.5		35	11	2.8
	84	7	1.8		52	10	2.5
	86	2	0.5		58	14	3.5
	87	0	0.0		67	15	3.8
	89	3	0.8		Total	128	32.2
	Total	61	15.3	A10	6	10	2.5
A5	26	4	1.0		11	4	1.0
	51	18	4.5		13	0	0.0
	69	0	0.0		44	2	0.5
	82	1	0.3		74	2	0.5
	Total	23	5.8		Total	18	4.5
A6	53	4	1.0	A11	73	8	2.0
	30	3	0.8	A13	54	8	2.0
	56	11	2.8	A15	71	0	0.0
	66	20	5.0		90	10	2.5
	Total	38	9.5		Total	10	2.5
A7	18	25	6.3	Total		399	
	39	10	2.5				
	45	13	3.3				
	59	10	2.5				
	68	1	0.3				
	70	13	3.3				
	85	3	0.8				
	Total	75	18.8				

HPV 16 and/or 18 were found in 2.7% of women with normal cytology, 27.3% of women with low-grade cervical lesions (ASCUS, LSIL) and in 52.4% of those with high-grade lesion (Table IV). HPV 16 and HPV 33 were the most frequent types detected in high-grade lesions.

**Table IV:** Prevalence of Group 1 HPV types by cytological outcome

	Normal		Low-grade		High-grade	
			
HPV type	n	%	n	%	n	%
16/18	33	2.7	27	27.3	11	52.4
16	23	1.9	16	16.2	10	47.6
18	10	0.8	14	14.1	1	4.8
31	7	0.6	2	2.0	2	9.5
33	10	0.8	5	5.1	3	14.3
35	7	0.6	2	2.0	2	9.5
39	7	0.6	3	3.0	0	0.0
45	10	0.8	3	3.0	0	0.0
51	12	1.0	5	5.1	1	4.8
52	4	0.3	5	5.1	1	4.8
56	7	0.6	4	4.0	0	0.0
58	7	0.6	7	7.1	0	0.0
59	7	0.6	2	2.0	0	0.0
Total	1,239		99		21	

## Discussion

The prevalence of HPV infection in the study participants (21.4%) is in the upper range of results that have been reported worldwide in women with normal cervical cytology (19). A study with a similar methodology in the United States showed a prevalence of 26.8% (20), while studies conducted in Canada reported HPV infection rates of 16.8% (British Columbia) (21), 13.3% (Ontario) (22), 28% (New-Brunswick) (23) and 19% (Manitoba) (24). A study conducted in a population of Inuit women living in northern Quebec reported a prevalence of 29% (6) and our own study in the Northwest Territory reported a prevalence of 24.2% (25). The prevalence of any cytological abnormality (8.8%) is also in keeping with the reported rates of cytological abnormalities in Canada (7% in Nunavik, Manitoba and British Columbia) (6,21,24).

HPV 16 was the most prevalent HPV type observed in the present study, which is similar to that previously reported worldwide (26); HPV 18 was the second most prevalent type. HPV31 has been found as the second most prevalent type in some areas of Canada (6), but not in Labrador. The prevalence of HPV 16 in high-grade cervical lesions (48%) was in the range of that reported worldwide (45%) (11), as well as in Canada (65%) (27). As expected, the prevalence of HPV infections was higher in younger women and decreased markedly with increasing age, which reflects the natural history of the infection (28).

The prevalence of multiple HPV infections in this study was 5.6%, or 26.2% of HPV-positive women. This proportion of multiple infections is in the middle of the worldwide range of 11.5–42.4% for women with a normal cervix (25). It is, however, lower than in other studies in Northern regions (see for example 40% relative prevalence in Nunavik, Quebec (6) or 45% in rural aboriginal women in the United States) (29). Detection of multiple infections may be influenced by the performance of the genotyping method. In comparison with the Roche LinearArray kit (a widely used HPV genotyping method), the Luminex method used in this study was shown to detect fewer HPV types in multiple infections with 3 or more types, but not fewer infected women (17). It is therefore possible that the percentage of multiple infections may be slightly, but not drastically, underestimated. A similar study by our group using the same Luminex method has detected 35.2% multiple infections in HPV-positive women (25).

Labrador has a predominantly Aboriginal population (Métis, Inuit, and First Nations), especially outside Happy Valley-Goose Bay. In the 2006 Canadian Census, 38% of the residents of Happy Valley-Goose Bay (the 95% of population of the Central Region) self-identified as Aboriginal, while at least 87% of the people living outside Happy Valley-Goose Bay self-identified as Aboriginal. Higher prevalence of HPV in Aboriginal groups living in Canada has previously been reported (6–8). No significant difference was found between the Labrador regions in terms of HPV infection rates or rates of cytological outcomes.

Our study shows that the distribution of HPV types in women undergoing routine cervical cancer screening in Labrador is in accordance with observations reported in Canada and worldwide. These results indicate that the current HPV vaccine against HPV 16 and 18 is likely to have the expected effectiveness in preventing cervical cancer in Labrador. A careful investigation of the underlying risk factors would help guide strategies for the prevention of cervical cancer in the population of Labrador.
